# Optimal doses of high-intensity interval training in patients with coronary artery disease and heart failure: a systematic review and meta-analysis

**DOI:** 10.3389/fcvm.2025.1698310

**Published:** 2026-01-02

**Authors:** Yue Wu, Hong Wang

**Affiliations:** 1 School of Graduate, Wuhan Sports University, Wuhan, China; 2 School of Wushu, Wuhan Sports University, Wuhan, China

**Keywords:** high-intensity interval training, coronary artery disease, heart failure, optimal doses, meta-analysis

## Abstract

**Objective:**

This systematic review and meta-analysis aimed to determine the optimal exercise dose parameters of High-Intensity Interval Training (HIIT)—including frequency, total training period, session duration, recovery intensity, and number of sessions—for patients with coronary artery disease (CAD) or heart failure (HF), and to evaluate its effects on peak oxygen uptake (VO_2_peak) and oxygen consumption at the first ventilatory threshold (VO_2_ at VT1).

**Methods:**

Randomized controlled trials (RCTs) investigating HIIT in CAD or HF patients and reporting VO_2_peak and/or VO_2_ at VT1 were identified by searching seven databases. The Cochrane RoB2 tool and RevMan 5.4 software were used for risk-of-bias assessment and statistical analysis.

**Results:**

Nineteen RCTs involving 1,152 patients (HIIT: *n* = 571; control: *n* = 581) were included. HIIT significantly improved VO_2_peak in both CAD patients (+1.69 mL·kg^−1^·min^−1^, 95% CI: 1.02–2.35, *P* < 0.00001) and HF patients (+2.46 mL·kg^−1^·min^−1^, 95% CI: 0.64–4.28, *P* = 0.008), with a greater improvement observed in HF. VO_2_ at VT1 also significantly increased in CAD (3 studies, *n* = 501: +0.97 mL·kg^−1^·min^−1^, 95% CI: 0.39–1.56, *P* = 0.001; *I*^2^ = 34%) and HF (5 studies, *n* = 112: +1.39 mL·kg^−1^·min^−1^, 95% CI: 0.23–2.56, *P* = 0.02; *I*^2^ = 0%). Subgroup analyses indicated that improvements in VO_2_peak were influenced by patient characteristics and exercise parameters. For CAD, greater benefits were associated with single-session duration >35 min, ≥36 sessions, and total training period ≥12 weeks. For HF, benefits were linked to single-session duration >35 min and frequency ≥3 days/week. Heterogeneity was moderate for CAD (*I*^2^ = 45%) and high for HF (*I*^2^ = 79%), suggesting variations related to study design and HIIT protocols.

**Conclusion:**

HIIT effectively improves VO_2_peak in both CAD and HF patients, with potentially greater benefits in HF. Dose-response analysis provides preliminary insights into its effect on submaximal exercise capacity (VO_2_ at VT1). Optimal parameters are: for CAD—frequency ≥2 days/week, total period ≥12 weeks, session duration >35 min, active recovery intensity ≥40% VO_2_peak, work/rest ratio 0.5–1.33; for HF—frequency ≥3 days/week, total period ≥12 weeks, session duration >35 min, active recovery intensity ≥40%, work/rest ratio 0.5–1.

## 1 Introduction

Coronary artery disease (CAD) is one of the leading cardiovascular causes of death worldwide. Epidemiological data indicate that CAD accounts for over 17.5 million deaths annually and ranks as the primary cause of mortality in both developed and developing countries (1). China likewise bears a substantial public health burden attributable to CAD. Numerous clinical studies have demonstrated that exercise-based cardiac rehabilitation, as a nonpharmacological intervention, plays a crucial role in the prevention and management of CAD (2). Peak oxygen uptake (VO_2_peak) is a key objective measure of cardiopulmonary function in patients with CAD; it has been widely shown to correlate strongly with all-cause mortality and serves as an important predictor of long-term prognosis (3). Evidence further suggests that high-intensity interval training (HIIT) can significantly increase VO_2_peak, thereby improving myocardial perfusion, promoting collateral circulation, and optimizing systemic metabolic function, ultimately helping to slow CAD progression and improve clinical outcomes (4).

Heart Failure (HF) is another common cardiovascular disease with a poor prognosis and one of the leading causes of death globally (5). Data shows that approximately 920,000 people in the UK suffer from HF (6). Patients often present with symptoms such as shortness of breath, fluid retention, fatigue, and a severe decline in exercise tolerance (7). These symptoms not only significantly affect the quality of life but are also important predictors of increased risk of readmission and death (8). Although drug therapy can relieve HF symptoms to some extent (9), its effectiveness is often limited and difficult to maintain in the long term. Exercise training, especially for patients with stable-stage HF, demonstrates unique rehabilitation value (10). A scientifically designed exercise program can significantly improve the patient's VO_2_peak, a core indicator reflecting cardiopulmonary function and exercise tolerance. Therefore, combining individualized exercise training with drug therapy is of great clinical significance for improving the prognosis of HF patients, reducing readmission, and enhancing the quality of life (11). In traditional studies, VO_2_peak has been widely used to evaluate the exercise tolerance and prognosis of heart failure patients. However, VO_2_peak may be limited by the patient's subjective effort and lacks sensitivity in reflecting the central physiological adaptations brought about by exercise training. In recent years, increasing evidence suggests that the oxygen uptake corresponding to the first ventilatory threshold is a more sensitive indicator than VO_2_peak, which can more effectively and earlier reveal the improvement in endurance capacity and exercise efficiency of heart failure patients (12). Therefore, this study aims to explore the impact of exercise-based rehabilitation training on heart failure patients, with a focus on its improvement effect on VO_2_ at VT1, and to evaluate its relationship with the changes in the traditional indicator VO_2_peak.

In recent years, high-intensity interval training (HIIT) has gradually become a research hotspot in the exercise rehabilitation of patients with coronary artery disease (CAD) and heart failure (HF) due to its excellent time efficiency and clinical effects. In well-trained athletes, high-intensity interval training (HIIT) is significantly superior to simply increasing the volume of moderate-intensity continuous training (MICT) in breaking through the plateau of endurance performance and can effectively improve indicators such as peak oxygen uptake (VO_2_peak) (13). However, the underlying physiological adaptation mechanisms, such as changes in skeletal muscle buffering capacity, still need in-depth exploration. The high-intensity interval training (HIIT) mode significantly reduces the risk of major adverse cardiovascular events through multiple mechanisms, such as improving cardiopulmonary fitness, enhancing myocardial perfusion, improving vascular endothelial function, and promoting metabolic adaptation (14). Despite the broad application prospects of HIIT, there is still no consensus on its optimal exercise dosage (including key parameters such as intensity, interval time, single duration, and total cycles) for different cardiovascular populations (15). Determining a scientific and effective HIIT dosage regimen is crucial for achieving personalized rehabilitation treatment and maximizing clinical benefits.

Therefore, this study used systematic review and Meta-analysis methods to comprehensively evaluate the efficacy and safety of high-intensity interval training (HIIT) in patients with coronary artery disease (CAD) and heart failure (HF), and focused on exploring its optimal exercise dosage parameters (including intensity, single duration, interval arrangement, and total cycles), aiming to provide theoretical basis and clinical practice guidance for formulating personalized exercise rehabilitation programs based on evidence-based medicine.

## 2 Materials and methods

This systematic review strictly adhered to the standard requirements of the PRISMA (Registration, Reporting, and Collaboration Mechanism for Randomized Controlled Trials) guidelines, ensuring the transparency and scientific rigor of the evaluation process. The research protocol has been officially registered in the internationally renowned PROSPERO database and has a unique registration number CRD420251128651.

### 2.1 Search strategy

All English publications published before September 2025 in databases such as MEDLINE, CINAHL, Web of Science, PubMed, Cochrane Library, Embase, and EBSCO were retrieved. The Medical Subject Headings (MeSH) database was used to identify all relevant articles regarding High-Intensity Interval Training (HIIT) and Coronary Artery Disease (CAD) or Heart Failure (HF). The MeSH terms used were “Coronary Artery Disease” OR “Heart Failure” OR “Cardiac Failure” OR “Heart Decompensation” AND “Randomized Controlled Trial” and their related terms. The text words used in combination with MeSH terms were “High-Intensity Interval Training” OR “Interval Training, High-Intensity” OR “Trainings, High-Intensity Interval”. The specific search strategy can be referred to in Supplementary Appendix S1.

### 2.2 Eligibility criteria

Our research team has formulated detailed inclusion and exclusion criteria. We included randomized controlled trials published in English before September 2025. These trials require the intervention of high-intensity interval training (HIIT) in patients with coronary artery disease (CAD) or heart failure (HF), and report peak oxygen uptake (VO_2_peak) and/or the first ventilation threshold as the primary or secondary outcome measure. Exclusion criteria include: mixed interventions combining HIIT with strength training, studies that did not independently report VO_2_peak or VO_2_ at VT1 outcome data, studies whose subjects included patients after heart transplantation, grafts, or valvular lesions, and studies using dietary supplements, special nutritional interventions, or drug-assisted tests.

### 2.3 Data extraction and management

In this study, the data extraction and management process was independently carried out by two researchers, who were responsible for systematically extracting and encoding relevant data from eligible studies to ensure the accuracy and consistency of the information. All extracted data is then reviewed by a third independent expert to verify its completeness, reliability, and compliance with the predetermined extraction specifications. The extracted content mainly includes the following aspects: Basic research information (such as title, publication year and first author), baseline characteristics of the subjects, type of heart failure (HFrEF or HFpEF), sample size, specific details of the HIIT intervention plan (such as intensity, duration, interval arrangement and total cycle) And related outcome indicators (such as VO_2_peak, changes in VO_2_ at VT1), etc.

### 2.4 Quality assessment

The quality assessment of the included studies was independently conducted by two reviewers using the Cochrane Risk of Bias Tool (RoB2). Reviewers conduct a systematic evaluation of the risk of bias for each study from multiple dimensions, such as trial design, implementation, and reporting, based on the signal questions provided by the tool. In case of differences of opinion, first reach a consensus through discussion. If a consensus still cannot be reached, a third judge will be invited to participate in the adjudication. The overall evidence confidence of each analysis result was classified into three levels: high, low, or unclear. The initial assessment is set at a high confidence level. Subsequently, for each area with a risk of bias, the confidence level is correspondingly lowered by one level.

### 2.5 Statistical analysis

In this study, Review Manager 5.4 software was used for statistical analysis based on the random-effects model and the fixed-effects model. The aim was to identify which disease groups (CAD or HF) would benefit most significantly from high-intensity interval training (HIIT) and to compare the differences in the effects of HIIT intervention between patients with CAD and HF. During the processes of literature retrieval, screening, and data extraction, we fully considered the key variables of the HIIT protocol, including the work/rest ratio, duration of the high-intensity phase, exercise intensity, and intervention frequency.

When dealing with inter-study heterogeneity, we first used the mean difference (MD) method to pool the data and employed a random-effects model to summarize the effect sizes. Heterogeneity was tested using the *I*^2^ statistic. To evaluate the stability and reliability of the results, we conducted a sensitivity analysis by excluding individual studies one by one to observe their impact on the overall effect. Meanwhile, we examined the role of continuous variables as potential moderators of VO_2_peak and divided the studies into subgroups based on the median of the continuous variables for further analysis. In the subgroup analysis, we explored the association between the changes in VO_2_peak before and after HIIT intervention and the changes in potential moderator variables. Additionally, to assess publication bias, we applied both the Begg's test and the Egger's test, where *P* > 0.05 indicated no significant publication bias. Sensitivity analysis was also included to comprehensively judge the robustness of the results.

## 3 Results

### 3.1 Search results

**Figure 1:**
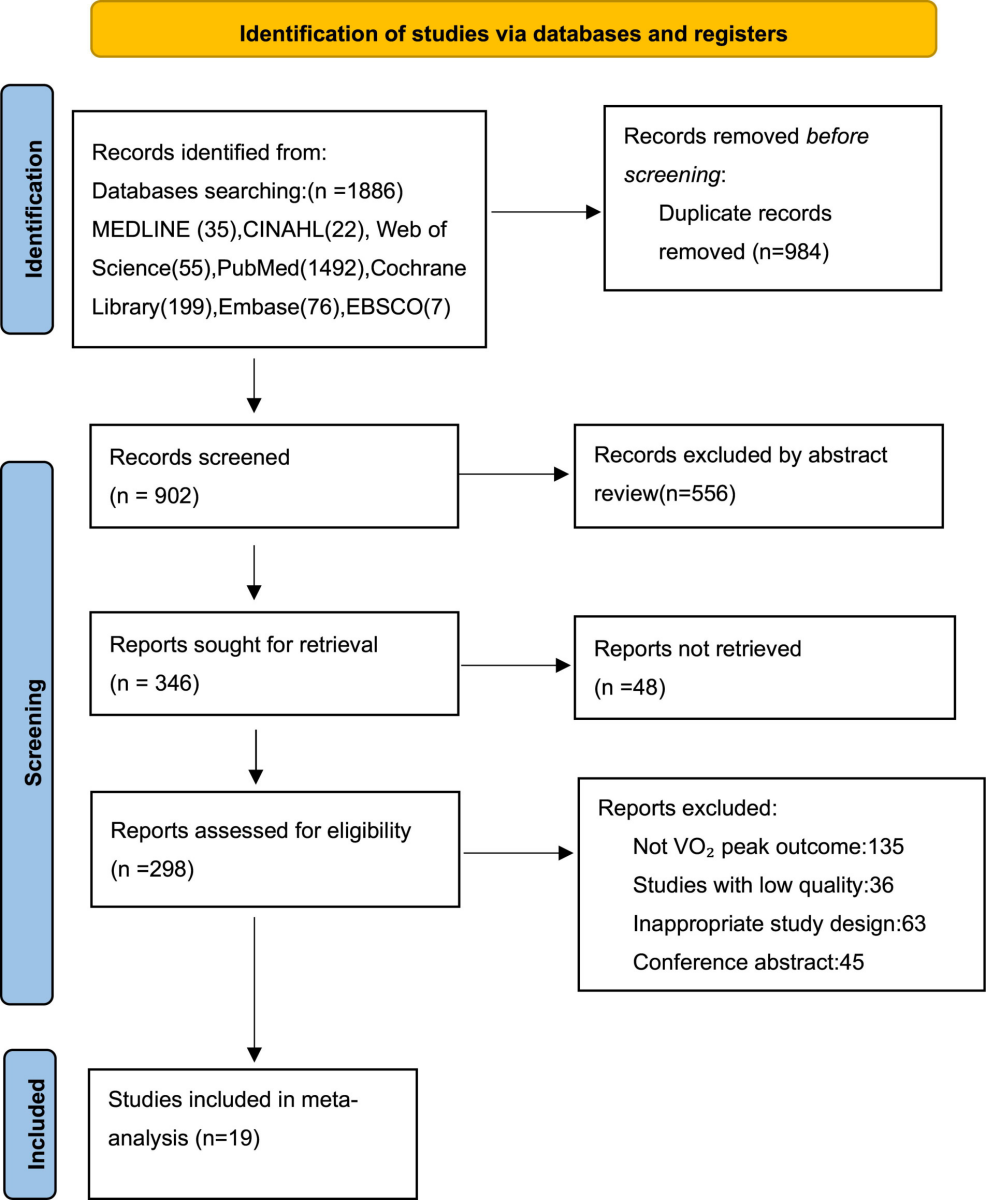
Flow diagram.

In this study, 1,886 research articles were retrieved from 7 databases. After removing the duplicate articles, 902 articles remained. Through further screening, 556 articles were found to be irrelevant to the research topic, and the remaining 346 articles needed to be read in full-text. According to the exclusion and inclusion criteria of this study, 19 studies were finally included (Figure 1).

### 3.2 Study characteristics

A total of 19 studies were included in this Meta-analysis, among which 9 focused on coronary artery disease (CAD) and 10 on heart failure (HF). A total of 1,152 patients were included, with 571 in the intervention group and 581 in the control group. In the studies of HF patients, patients with HFrEF accounted for 54.6% (183/335), and those with HFpEF accounted for 45.4% (152/335). One study included a mixed population and was therefore not included in this proportional analysis. The basic characteristics of the included studies are shown in Table 1, and the training characteristics of high-intensity interval training (HIIT) are shown in Table 2.

**Table 1:** General characteristics of the studies included in the meta-analysis.

Research studies	Study location	Disease	n-HIIT	HIIT (Male/female)	HIIT (Age, year)	HIIT (BMI, cm/kg^2^)
S. Mueller et al. (16)	Berlin, Leipzig, Munich, Belgium	HFpEF	58	17/41	70 ± 7	30.0 ± 5.7
R. F. Spee et al. (17)	Netherlands	HFrEF	12	10/2	58 ± 7.8	
N. M. Benda et al. (18)	Netherlands	HFrEF	10	9/1	63 ± 8	28.1 ± 7.5
R. F. Spee et al. (19)	Netherlands	HFrEF	12	12/0	68.9 ± 6.7	
C. H. Chou et al. (20)	Taiwan	HFrEF, HFpEF	17	12/5	61.2 ± 0.9	
A.D. da Silveira et al. (21)	Brazil	HFpEF	10	7/3	60 ± 10	33.5 ± 1.3
N. Turri-Silva et al. (22)	Brazil	HFrEF	8	5/3	60.9 ± 9.7	24.9 ± 5.2
S. S. Angadi et al. (23)	Arizona, USA	HFpEF	9	8/1	69.0 ± 6.1	29.8 ± 5.1
F. Besnier et al. (24)	Toulouse, France	HFrEF	16	11/5	59.5 ± 12	28 ± 5
S. C. Huang et al. (25)	Taiwan	HFrEF	33	26/7	60 ± 3	
K.V. Jaureguizar et al. (26)	Spain	CAD	36	28/8	58 ± 11	29.5 ± 4.1
G. G. Cardozo et al. (27)	Rio de Janeiro, Brazil	CAD	23	14/9	56 ± 12	27.5 ± 5.9
K. D. Currie et al. (28)	Canada	CAD	9	9/0	63 ± 8	28.9 ± 4.8
G. McGregor et al. (29)	United Kingdom	CAD	187	176/11	58.6 ± 9.2	29.1 ± 4.5
E. K. Vesterbekkmo et al. (30)	Norway	CAD	29	27/2	57.3 ± 6.8	28.9 ± 4
C. Gonçalves et al. (31)	Portugal	CAD	23	20/3	50 ± 9	28.2 ± 4.5
K. D. Currie et al. (32)	Canada	CAD	11	10/1	62 ± 11	27.9 ± 4.9
S. Khadanga et al. (33)	Vermont, USA	CAD	22	0/22	64.8 ± 9.0	29.4 ± 7
J. L. Taylor et al. (34)	Brisbane, Australia	CAD	46	39/7	65 ± 7	28.2 ± 4.2

BMI, body mass index; CAD, coronary artery disease; HF, heart failure; n-HIIT, number of participants in high-intensity interval training groups. Data are presented as mean, mean ± standard deviation, range, or number.

**Table 2:** Characteristics of high-intensity interval training in the studies.

Studies	Disease	HIIT Type	Frequency (days/week)	Session duration (min)	Interval work (min)	Interval recovery (min)	Duration (weeks)	Number of sessions (weeks)	Work intensity (% VO_2_peak)	*R* (work/recovery)
S. Mueller et al. (16)	HFpEF	HIII-L	3	38	4	3	12	36	80–90	1.33
R. F. Spee et al. (17)	HFrEF	HIIT-L	3	35	4	3	12	36	85–95	1.33
N. M. Benda et al. (18)	HFrEF	HIIT-S	2	35	1	2.5	12	24	90	0.83
R. F. Spee et al. (19)	HFrEF	HIIT-L	3	35	4	3	12	36	85–95	1.33
C. H. Chou et al. (20)	HFrEF, HFpEF	HIIT-L	3	30	3	3	12	36	80	1
A.D. da Silveira et al. (21)	HFpEF	HIIT-L	3	38	4	3	12	36	80–90	1.33
N. Turri-Silva et al. (22)	HFrEF	HIIT-L	3	50	3	4	12	36	75–85	0.75
S. S. Angadi et al. (23)	HFpEF	HIIT-L	3	45	4	3	4	12	85–90	1.33
F. Besnier et al. (24)	HFrEF	HIIT-S	5	20	0.5	0.5	3.5	19	100	1
S. C. Huang et al. (25)	HFrEF	HIIT-L	3	50	3	3	12	36	80	1
K.V. Jaureguizar et al. (26)	CAD	HIIT-S	3	40	0.33	0.67	8	24	85	0.5
G. G. Cardozo et al. (27)	CAD	HIIT-L	3	40	2	2	16	48	90	1
K. D. Currie et al. (28)	CAD	HIIT-S	2	20	1	1	24	48	85	1
G. McGregor et al. (29)	CAD	HIIT-S	2	20	1	1	8	16	85–90	1
E.K.Vesterbekkmo et al. (30)	CAD	HIIT-L	2	40	4	3	24	48	85–95	1.33
C. Gonçalves et al. (31)	CAD	HIIT-S	3	35	4	1	6	18	85–95	4
K. D. Currie et al. (32)	CAD	HIIT-S	3	20	1	1	12	36	90	1
S. Khadanga et al. (33)	CAD	HIIT-L	3	33	4	4	12	36	90–95	1
J. L. Taylor et al. (34)	CAD	HIIT-L	3	25	4	3	4	12	87	1.33

CAD, coronary artery disease; HF, heart failure; HIIT, high-intensity interval training; HIIT-L, protocol with long work intervals; HIIT-S, protocol with short work intervals; R, work/recovery ratio; % VO_2_peak, percentage of peak oxygen uptake during work interval.

### 3.3 Risk-of-bias assessment

In this study, the RoB2 tool was used to evaluate the quality of 19 RCTs. The results showed that the included studies performed well in terms of randomization and data integrity, but there were limitations in the implementation of blinding, which was consistent with the characteristics of exercise intervention studies.

Specifically, in terms of Random sequence generation, 16 studies were at low risk, and 3 were of unclear risk due to unclear descriptions. Regarding Allocation concealment, 12 studies were at low risk, 5 were of unclear risk, and 2 were at high risk. Affected by the characteristics of exercise intervention, the blinding of participants and personnel in 15 studies was at high risk, and 4 were of unclear risk. In terms of blinding of outcome assessment, 9 studies were at low risk, 6 were at high risk, and 4 were of unclear risk. For incomplete data, 17 studies were at low risk, and 2 were at high risk. In terms of selective reporting, 16 studies were at low risk, and 3 were of unclear risk. No other obvious biases were found in all the studies.

**Figure 2:**
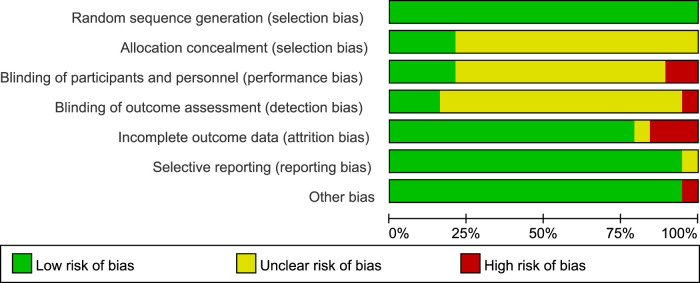
Distribution of the percentage of literature bias.

**Figure 3:**
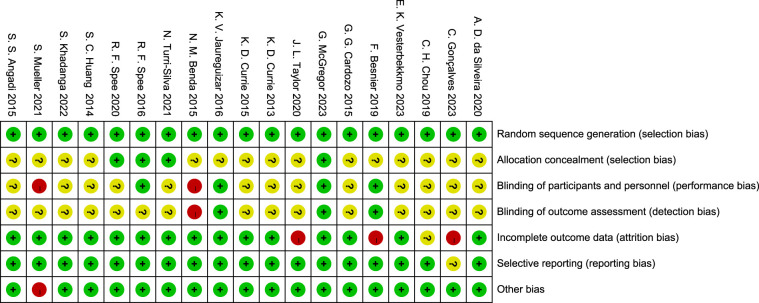
Analysis of the risk of literature bias.

In conclusion, despite the limitations of blinding, the overall quality of the included studies is good in terms of randomization, data integrity, and reporting transparency, providing a reliable basis for the results of the Meta-analysis (Figures 2, 3).

## 4 Meta-analysis results

### 4.1 Effects of high-intensity interval training on VO_2_peak in patients with coronary artery disease and heart failure

**Figure 4:**
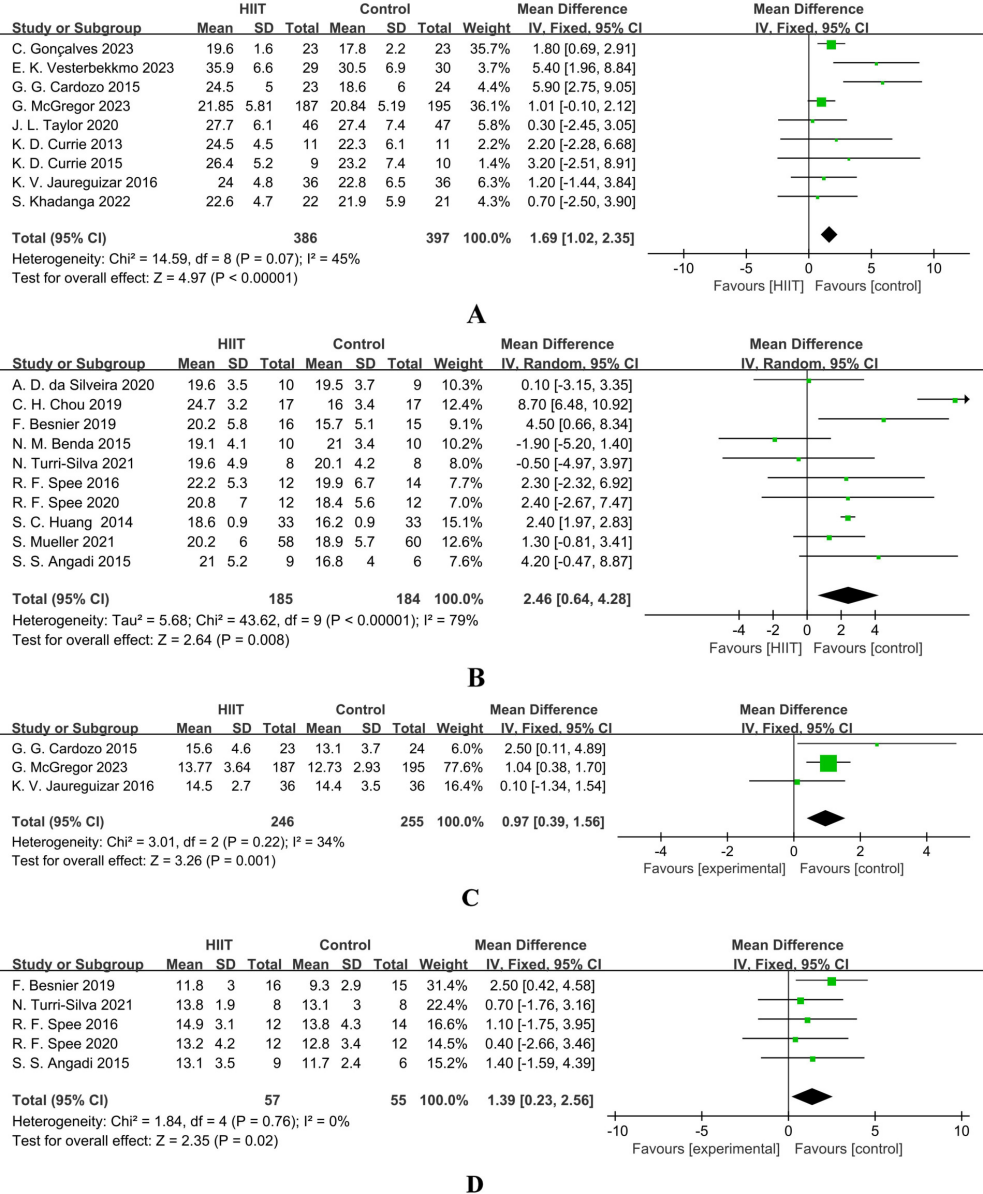
**(A)** forest plot showing the mean difference (MD) in peak VO_2_ (mL·kg^−1^·min^−1^) between HIIT and control groups in patients with coronary artery disease. **Positive values favour HIIT**. **(B)** Forest plot showing the mean difference (MD) in peak VO_2_ (mL·kg^−1^·min^−1^) between HIIT and control groups in patients with heart failure. **Positive values favour HIIT**. **(C)** Forest plot showing the mean difference (MD) in VO_2_ at first ventilatory threshold (VO_2_ AT VT1, mL·kg^−1^·min^−1^) between high-intensity interval training (HIIT) and control groups in patients with coronary artery disease. **Positive values favour HIIT**. **(D)** Forest plot showing the mean difference (MD) in VO_2_ at first ventilatory threshold (VO_2_ AT VT1, mL·kg^−1^·min^−1^) between high-intensity interval training (HIIT) and control groups in patients with heart failure. **Positive values favour HIIT**.

Figure 4A presents the results of a Meta-analysis on the effect of HIIT on VO_2_peak in patients with coronary artery disease (CAD). A total of 9 studies were included (386 in the intervention group and 397 in the control group). The pooled analysis showed that HIIT could significantly increase VO_2_peak [MD = 1.69, 95% CI (1.02, 2.35), *Z* = 4.97, *P* < 0.00001], suggesting that HIIT can effectively improve the cardiopulmonary function of CAD patients. The heterogeneity was moderate (*I*^2^ = 45%, *P* = 0.07), and a fixed-effect model was used.

Figure 4B presents the results of a Meta-analysis on the effect of HIIT on VO_2_peak in patients with heart failure (HF). A total of 10 studies were included (185 in the intervention group and 184 in the control group). The results showed a more significant increase in VO_2_peak [MD = 2.46, 95% CI (0.64, 4.28), Z = 2.64, *P* = 0.008], but there was extremely high heterogeneity (*I*^2^ = 79%, *P* < 0.00001), so a random-effects model was used. This indicates that HIIT is generally effective for HF patients, but the effect is influenced by factors such as study design and protocol differences, and individualized adjustment of the intervention protocol is required.

### 4.2 Effects of high-intensity interval training on VO_2_ at ventilatory threshold (VO_2_ AT VT1) in patients with coronary artery disease and heart failure

In patients with CAD, only 3 studies reported VO_2_ at VT1. The meta-analysis (Figure 4C) showed that HIIT had a tendency to increase it [MD = 0.97, 95% CI (0.39, 1.56), *Z* = 3.26, *P* = 0.001], and the result was statistically significant (*Z* = 3.26, *P* = 0.001). However, due to the limited number of studies that could be combined, the interpretation of the results still needs to be cautious. The heterogeneity was mild (*I*^2^ = 34%), and subgroup analysis was not performed due to the insufficient number of studies.

In patients with heart failure (HF), an analysis of five studies (Figure 4D) indicated that high-intensity interval training (HIIT) could significantly improve VO_2_ at the first ventilatory threshold (VT1) [MD = 1.39, 95% CI (0.23, 2.56), *Z* = 2.35, *Z* = 2.35, *P* = 0.02], with low heterogeneity (*I*^2^ = 0%), supporting its use as a sensitive indicator for evaluating the improvement of endurance in HF patients.

### 4.3 Comparative analysis of HIIT-induced changes in VO_2_peak and VO_2_ at VT1

This study compared the effects of HIIT on VO_2_peak and VO_2_ at VT1 to evaluate their sensitivities in detecting endurance improvement.

In patients with heart failure, HIIT significantly improved VO_2_peak (MD = 2.46) and VO_2_ at VT1 (MD = 1.39). Although the absolute improvement in VO_2_peak was greater, VO_2_ at VT1 had extremely low heterogeneity (*I*^2^ = 0%), in sharp contrast to the extremely high heterogeneity of VO_2_peak (*I*^2^ = 79%), indicating that VO_2_ at VT1 is a more robust indicator of physiological adaptation across studies.

A similar trend was also observed in patients with coronary artery disease: HIIT significantly improved both VO_2_peak (MD = 1.69) and VO_2_ at VT1 (MD = 0.97), and the heterogeneity of VO_2_ at VT1 (*I*^2^ = 34%) was lower than that of VO_2_peak (*I*^2^ = 45%), supporting the view that VO_2_ at VT1 is a more stable measurement indicator.

In conclusion, although HIIT effectively improves the maximum exercise capacity, its effect on the submaximal threshold is a more consistent finding. The low heterogeneity of the VO_2_ at VT1 response supports its use as the primary endpoint for evaluating the improvement of functional endurance in cardiac patients.

### 4.4 VO_2_peak was analyzed across subgroups based on population characteristics

Based on the results of subgroup analysis (Tables 3, 4), this study systematically evaluated the potential moderating effects of different population characteristics on the efficacy of high-intensity interval training (HIIT) in improving peak oxygen uptake (VO_2_peak) in patients with coronary artery disease (CAD) and heart failure (HF). The differences among subgroups were tested by an interaction test (Chi-square test), and a significance level was set at *P* < 0.05.

**Table 3:** Subgroup analyses assessing potential moderating factors for VO_2_peak increase in studies on CAD disease included in the meta-analysis by population characteristics.

	Research studies		VO_2_peak			
Group	CAD groups	References	MD (95% CI)	*I*^2^ (%)	*P* ^b^	Test for subgroup differences
No. of participants^a^						
<14	2	K. D. Currie et al. (32), K. D. Currie et al. (28)	2.58[−0.94,6.11]	0	=0.15	Chi^2^ = 0.26, df = 1 (*P* = 0.61)
≥14	7	C. Gonçalves et al. (31), E. K. Vesterbekkmo et al. (30), G. G. Cardozo et al. (27), G. McGregor et al. (29), J. L. Taylor et al. (34), K. V. Jaureguzar et al. (26), S. Khadanga et al. (33)	1.65[0.98,2.33]	58	<0.00001	
Age, year						
>60	4	J. L. Taylor et al. (34), K. D. Currie et al. (32), K. D. Currie et al. (28), S. Khadanga et al. (33),	1.02 [−0.78, 2.81]	0	=0.27	Chi^2^ = 0.62, df = 1 (*P* = 0.43)
≤60	5	C. Gonçalves et al. (31), E. K. Vesterbekkmo et al. (30), G. G. Cardozo et al. (27), G. McGregor et al. (29), K. V. Jaureguzar et al. (26)	1.79 [1.08, 2.51]	69	<0.00001	
Methodological quality						
>7 points	4	E. K. Vesterbekkmo et al. (30), G. McGregor et al. (29), J. L. Taylor et al. (34), K. V. Jaureguzar et al. (26)	1.26 [0.34, 2.18]	52	=0.007	Chi^2^ = 1.67, df = 1 (*P* = 0.20)
≤7 points	5	C. Gonçalves et al. (31), G. G. Cardozo et al. (27), K. D. Currie et al. (32), K. D. Currie et al. (28), S. Khadanga et al. (33)	2.14 [1.18, 3.10]	41	<0.0001	

CAD groups, coronary artery disease groups; 95% CI, 95% confidence interval; *I*^2^, heterogeneity; MD, mean difference; VO_2_peak, peak oxygen uptake.

Certain enrolled studies were not included because the value used for subgroup analysis was not reported in them.

a Number of subjects in the CAD group.

b Test for overall effect.

**Table 4:** Subgroup analyses assessing potential moderating factors for VO_2_peak increase in studies on HF disease included in the meta-analysis by population characteristics.

	Research studies		VO_2_peak			
Group	HF groups	References	MD (95% CI)	*I*^2^ (%)	*P* ^b^	Test for subgroup differences
No. of participants^a^						
<14	6	A. D. da Silveira et al. (21), N. M. Benda et al. (18), N. Turri-Silva et al. (22), R. F. Spee et al. (17), R. F. Spee et al. (19), S. S. Angadi et al. (23)	0.86 [−1.67, 3.39]	14	=0.51	Chi^2^ = 6.01, df = 1 (*P* = 0.01)
≥14	4	C. H. Chou et al. (20), F. Besnier et al. (24), S. C. Huang et al. (25), S. Mueller et al. (16)	2.55 [2.15, 2.96]	90	<0.00001	
Age, year						
>60	7	C. H. Chou et al. (20), N. M. Benda et al. (18), N. Turri-Silva et al. (22), R. F. Spee et al. (19), S. C. Huang et al. (25), S. Mueller et al. (16), S. S. Angadi et al. (23)	2.40 [2.00, 2.80]	85	<0.00001	Chi^2^ = 0.18, df = 1 (*P* = 0.67)
≤60	3	A. D. da Silveira et al. (21), F. Besnier et al. (24), R. F. Spee et al. (17)	2.37 [−0.20, 4.94]	32	=0.07	
Methodological quality						
>7 points	4	N. Turri-Silva et al. (22), R. F. Spee et al. (17), R. F. Spee et al. (19), S. Mueller et al. (16)	1.48 [−0.45, 3.41]	0	=0.13	Chi^2^ = 2.04, df = 1 (*P* = 0.15)
≤7 points	6	A. D. da Silveira et al. (21), C. H. Chou et al. (20), F. Besnier et al. (24), N. M. Benda et al. (18), S. C. Huang et al. (25), S. S. Angadi et al. (23)	2.64 [2.21, 3.07]	88	<0.00001	

HF groups, heart failure disease groups; 95% CI, 95% confidence interval; *I*^2^, heterogeneity; MD, mean difference; VO_2_peak, peak oxygen uptake.

Certain enrolled studies were not included because the value used for subgroup analysis was not reported in them.

a Number of subjects in the HF group.

b Test for overall effect.

In patients with CAD, studies with a sample size of ≥14 showed a significant improvement in VO_2_peak (MD = 1.65, 95% CI [0.98, 2.33], p 60 years did not reach significance [MD = 1.02, 95% CI (–0.78, 2.81), *P* = 0.27], but the difference between groups was not statistically significant (Chi^2^ = 0.62, *P* = 0.43). Studies with a methodological quality score of ≤7 had a larger effect size (MD = 2.14, 95% CI [1.18, 3.10], p 7 still had a significant but smaller effect size [MD = 1.26, 95% CI (0.34, 2.18), *P* = 0.007], with no significant difference between groups (Chi^2^ = 1.67, *P* = 0.20).

In patients with HF, studies with a sample size of ≥14 showed a significant improvement in VO_2_peak (MD = 2.50, 95% CI [2.15, 2.96], p 60 years (MD = 2.40, 95% CI [2.00, 2.80], p 7 did not show a significant effect size [MD = 1.48, 95% CI (–0.45, 3.41), *P* = 0.13], and the difference between groups did not reach significance (Chi^2^ = 2.04, *P* = 0.1).

In conclusion, the improvement effect of HIIT on VO_2_peak in patients with CAD and HF is modulated by sample size, age, and study quality, with the influence of sample size being particularly significant. In patients with CAD, HIIT shows a more stable effect in elderly patients. However, since age-related differences may be affected by peak RER, meta-regression analysis is required for further verification. These results suggest that patient characteristics and study design should be comprehensively considered in clinical practice and future research to optimize individualized cardiac rehabilitation strategies.

### 4.5 VO_2_peak was analyzed across subgroups based on exercise characteristics

This study investigated the effects of different subgroups with distinct exercise characteristics on the improvement of peak oxygen uptake (VO_2_peak) in patients with coronary artery disease (CAD) and heart failure (HF). The key parameters were defined as follows: Interval Duration refers to the duration of a single work period or recovery period; Single-Session Duration is the total time of a complete high-intensity interval training (HIIT) session; Total Training Period represents the duration of the entire intervention study, usually measured in weeks or months. Analysis of variance (ANOVA) was used to test the interactions of differences among subgroups, and the significance level was set at *P* < 0.05.

In patients with CAD, the improvement in VO_2_peak was more significant in the group with Number of sessions ≥36 [MD = 3.4, 95% CI (1.90, 5.06), *P* < 0.0001], while the improvement was smaller in the group with <36 sessions [MD = 1.01, 95% CI (0.48, 1.54), *P* = 0.0004], and the difference between the groups was significant (Chi^2^ = 6.40, *P* = 0.01). The improvement was more obvious in the group with Total Training Perio ≥12 weeks [MD = 3.65, 95% CI (1.99, 5.30), *P* < 0.0001], and smaller in the group with <12 weeks [MD = 1.31, 95% CI (0.59, 2.04), *P* = 0.0004], with a significant difference between the groups (Chi^2^ = 6.40, *P* = 0.01). The improvement was more significant in the group with Single—session Duration >35 min [MD = 3.83, 95% CI (2.05, 5.61), *P* < 0.0001], and lower in the group with ≤35 min [MD = 1.22, 95% CI (0.54, 1.90), *P* = 0.0003], and the difference between the groups was significant (Chi^2^ = 6.09, *P* = 0.01). In the HIIT Protocol, the improvement was greater in the HIIT-L group [MD = 3.03, 95% CI (1.48, 4.59), *P* = 0.0001] and smaller in the HIIT-S group [MD = 1.44, 95% CI (0.70, 2.17), *P* = 0.0001], but the difference between the groups was not significant (Chi^2^ = 3.37, *P* = 0.07). There were no significant differences between subgroups in the subgroup analyses of HIIT frequency, Interval Duration, Interval recovery, Active recovery intensity, and work-recovery ratio (W/R) (*P* > 0.05) (Table 5).

**Table 5:** Subgroup analyses assessing potential moderating factors for VO_2_peak increase in studies on CAD disease included in the meta-analysis by exercise characteristics.

	Research studies		VO_2_peak			
Group	CAD groups	References	MD (95% CI)	*I*^2^ (%)	*P* ^a^	Test for subgroup differences
Number of sessions						
≥36 sessions	5	E. K. Vesterbekkmo et al. (30), G. G. Cardozo et al. (27), K. D. Currie et al. (32), K. D. Currieet al. (28), S. Khadanga et al. (33)	3.48 [1.90, 5.06]	40	<0.0001	Chi^2^ = 6.40, df = 1 (*P* = 0.01)
<36 sessions	4	C. Gonçalves et al. (31), G. McGregor et al. (29), J. L. Taylor et al. (34), K. V. Jaureguzar et al. (26)	1.01 [0.48, 1.54]	0	=0.0004	
Total Training Period						
≥12weeks	5	E. K. Vesterbekkmo et al. (30), G. G. Cardozo et al. (27), K. D. Currieet al. (32), K. D. Currieet al. (28), S. Khadanga et al. (33)	3.65 [1.99, 5.31]	40	<0.0001	Chi^2^ = 6.40, df = 1 (*P* = 0.01)
<12weeks	4	C. Gonçalves et al. (31), G. McGregor et al. (29), J. L. Taylor et al. (34), K. V. Jaureguzar et al. (26)	1.31 [0.59, 2.04]	0	=0.0004	
HIIT frequency						
3 or 4 days/week	6	C. Gonçalves et al. (31), G. G. Cardozo et al. (27), J. L. Taylor et al. (34), K. D. Currie et al. (32), K. V. Jaureguzar et al. (26), S. Khadanga et al. (33)	1.69 [0.90, 2.48]	40	<0.0001	Chi^2^ = 0.26, df = 1 (*P* = 0.61)
≤2 days/week	3	E. K. Vesterbekkmo et al. (30), G. McGregor et al. (29), K. D. Currieet al. (28)	1.69 [0.51, 2.87]	67	=0.005	
Single-session Duration						
>35 min	3	E. K. Vesterbekkmo et al. (30), G. G. Cardozo et al. (27), K. V. Jaureguzar et al. (26)	3.83 [2.05, 5.61]	68	<0.0001	Chi^2^ = 6.09, df = 1 (*P* = 0.01)
≤35 min	6	C. Gonçalves et al. (31), G. McGregor et al. (29), J. L. Taylor et al. (34), K. D. Currie et al. (32), K. D. Currie et al. (28), S. Khadanga et al. (33)	1.22 [0.54, 1.90]	0	=0.0003	
Interval duration						
>2.5 min	4	C. Gonçalves et al. (31), E. K. Vesterbekkmo et al. (30), J. L. Taylor et al. (34), S. Khadanga et al. (33)	1.80 [0.85, 2.74]	48	=0.0002	Chi^2^ = 0.11, df = 1 (*P* = 0.74)
≤2.5 min	5	G. G. Cardozo et al. (27), G. McGregor et al. (29), K. D. Currie et al. (32), K. D. Currie et al. (28), K. V. Jaureguzar et al. (26)	1.58 [0.64, 2.51]	54	=0.0010	
Interval recovery						
>2.5 min	3	E. K. Vesterbekkmo et al. (30), J. L. Taylor et al. (34), S. Khadanga et al. (33)	1.79 [0.01, 3.58]	65	=0.05	Chi^2^ = 0.02, df = 1 (*P* = 0.90)
≤2.5 min	6	C. Gonçalves et al. (31), G. G. Cardozo et al. (27), G. McGregor et al. (29), K. D. Currie et al. (32), K. D. Currie et al. (28), K. V. Jaureguzar et al. (26)	1.69 [0.96, 2.42]	43	<0.00001	
HIIT Protocol						
HIIT-L	4	E. K. Vesterbekkmo et al. (30), G. G. Cardozo et al. (27), J. L. Taylor et al. (34), S. Khadanga et al. (33)	3.03 [1.48, 4.59]	78	=0.0001	Chi^2^ = 3.37, df = 1 (*P* = 0.07)
HIIT-S	5	C. Gonçalves et al. (31), G. McGregor et al. (29), K. D. Currie et al. (32), K. D. Currie et al. (28), K. V. Jaureguizar et al. (26)	1.44 [0.70, 2.17]	0	=0.0001	
Active recovery intensity						
>40%	2	E. K. Vesterbekkmo et al. (30), S. Khadanga et al. (33)	2.88 [0.53, 5.22]	74	= 0.02	Chi^2^ = 1.08, df = 1 (*P* = 0.30)
≤40%	7	C. Gonçalves et al. (31), G. G. Cardozo et al. (27), G. McGregor et al. (29), J. L. Taylor et al. (34), K. D. Currie et al. (32), K. D. Currie et al. (28), K. V. Jaureguzar et al. (26)	1.58 [0.89, 2.28]	38	<0.00001	
R (W/R)						
>1	3	C. Gonçalves et al. (31), E. K. Vesterbekkmo et al. (30), J. L. Taylor et al. (34)	1.90 [0.92, 2.89]	62	= 0.0002	Chi^2^ = 0.26, df = 1 (*P* = 0.61)
≤1	6	G. G. Cardozo et al. (27), G. McGregor et al. (29), K. D. Currie et al. (32), K. D. Currie et al. (28), K. V. Jaureguzar et al. (26), S. Khadanga et al. (33)	1.58 [0.62, 2.54]	51	=0.001	

CAD groups, coronary artery disease groups; HIIT, high-intensity interval training; HIIT-L, protocol of HIIT with long work interval; HIIT-S, protocol of HIIT with short work interval; *I^2^*, heterogeneity; MD, mean difference; VO_2_peak: peak oxygen uptake; R (W/R), work-recovery ratio.

Certain studies were not included because they did not report the value used for subgroup analysis.

a Test for overall effect.

In patients with HF, the improvement of VO_2_peak in the group with Number of sessions ≥36 was significant [MD = 2.40, 95% CI (2.00, 2.80), *P* < 0.00001], but there was no significant difference compared with the group with <36 sessions (Chi^2^ = 0.73, *P* = 0.39). There were no statistically significant differences among groups in Total Training Period, HIIT frequency, Interval Duration, Interval recovery, HIIT Protocol (HIIT-L vs. HIIT-S), recovery mode, recovery intensity, and work-recovery ratio (*P* > 0.05). Single-session duration is a key factor influencing the improvement of VO_2_peak (Table 6).

**Table 6:** Subgroup analyses assessing potential moderating factors for VO_2_peak increase in studies on HF disease included in the meta-analysis by exercise characteristics.

	Research studies		VO_2_peak			
Group	HF groups	References	MD (95% CI)	*I*^2^ (%)	*P* ^a^	Test for subgroup differences
Number of sessions						
≥36 sessions	7	A. D. da Silveira et al. (21), C. H. Chou et al. (20), N. Turri-Silva et al. (22), R. F. Spee et al. (17), R. F. Spee et al. (19), S. C. Huang et al. (25), S. Mueller et al. (16)	2.40 [2.00, 2.80]	83	<0.00001	Chi^2^ = 0.73, df = 1 (*P* = 0.39)
<36 sessions	3	F. Besnier et al. (24), N. M. Benda et al. (18), S. S. Angadi et al. (23)	2.37 [−1.13, 5.87]	74	=0.18	
Total Training Period						
≥12 weeks	8	A. D. da Silveira et al. (21), C. H. Chou et al. (20), N. M. Benda et al. (18), N. Turri-Silva et al. (22), R. F. Spee et al. (17), R. F. Spee et al. (19), S. C. Huang et al. (25), S. Mueller et al. (16)	2.40 [2.00, 2.80]	83	<0.00001	Chi^2^ = 1.60, df = 1 (*P* = 0.21)
<12 weeks	2	F. Besnier et al. (24), S. S. Angadi et al. (23)	4.38 [1.41, 7.35]	0	=0.004	
HIIT frequency						
≥5 days/week	1	F. Besnier et al. (24)	4.50 [0.66, 8.34]	Not applicable	=0.02	Chi^2^ = 4.00, df = 2 (*P* = 0.14)
3 or 4 days/week	7	A. D. da Silveira et al. (21), C. H. Chou et al. (20), N. Turri-Silvaet al. (22), R. F. Spee et al. (17), R. F. Spee et al. (19), S. C. Huang et al. (25), S. Mueller et al. (16),	2.40 [2.00, 2.80]	83	<0.00001	
≤2 days/week	2	N. M. Benda et al. (18), S. S. Angadi et al. (23)	0.69 [−13.27, 14.65]	77	=0.92	
Single-session Duration						
>35 min	5	A. D. da Silveira et al. (21), N. Turri-Silvaet al. (22), S. C. Huang et al. (25), S. Mueller et al. (16), S. S. Angadi et al. (23)	2.26 [1.85, 2.67]	19	<0.00001	Chi^2^ = 8.98, df = 1 (*P* = 0.003)
≤35 min	5	C. H. Chou et al. (20), F. Besnier et al. (24), N. M. Benda et al. (18), R. F. Spee et al. (17), R. F. Spee et al. (19)	3.78 [2.53, 5.03]	87	<0.00001	
Interval duration						
>2.5 min	8	A. D. da Silveira et al. (21), C. H. Chou et al. (20), N. Turri-Silva et al. (22), R. F. Spee et al. (17), R. F. Spee et al. (19), S. C. Huang et al. (25), S. Mueller et al. (16), S. S. Angadi et al. (23)	2.50 [2.09, 2.90]	78	<0.00001	Chi^2^ = 1.68, df = 1 (*P* = 0.20)
≤2.5 min	2	F. Besnier et al. (24), N. M. Benda et al. (18)	1.30 [−2.63, 5.23]	84	0.52	
Interval recovery						
>2.5 min	8	A. D. da Silveira et al. (21), C. H. Chou et al. (20), N. Turri-Silva et al. (22), R. F. Spee et al. (17), R. F. Spee et al. (19), S. C. Huang et al. (25), S. Mueller et al. (16), S. S. Angadi et al. (23)	2.50 [2.10, 2.90]	80	<0.00001	Chi^2^ = 1.73, df = 1 (*P* = 0.19)
≤2.5 min	2	F. Besnier et al. (24), N. M. Benda et al. (18)	1.30 [−2.63, 5.23]	84	=0.52	
HIIT Protocol						
HIIT-L	8	A. D. da Silveira et al. (21), C. H. Chou et al. (20), N. Turri-Silva et al. (22), R. F. Spee et al. (17), R. F. Spee et al. (19), S. C. Huang et al. (25), S. Mueller et al. (16), S. S. Angadi et al. (23)	2.50 [2.10, 2.90]	80	<0.00001	Chi^2^ = 1.73, df = 1 (*P* = 0.19)
HIIT-S	2	F. Besnier et al. (24), N. M. Benda et al. (18)	1.30 [−2.63, 5.23]	84	=0.52	
Type of recovery						
Active (≥20%)	9	A. D. da Silveira et al. (21), A. D. da Silveira et al. (21), N. M. Benda et al. (18), N. Turri-Silva et al. (22), R. F. Spee et al. (17), R. F. Spee et al. (19), S. C. Huang et al. (25), S. Mueller et al. (16), S. S. Angadi et al. (23)	2.40 [2.00, 2.80]	81	<0.00001	Chi^2^ = 1.08, df = 1 (*P* = 0.30)
Passive (<20%)	1	F. Besnier et al. (24)	4.50 [0.66, 8.34]	Not applicable	=0.02	
Active recovery intensity						
>40%	4	A. D. da Silveira et al. (21), N. Turri-Silva et al. (22), S. Mueller et al. (16), S. S. Angadi et al. (23)	1.23 [−0.45, 2.91]	0	=0.15	Chi^2^ = 3.02, df = 1 (*P* = 0.08)
≤40%	5	C. H. Chou et al. (20), N. M. Benda et al. (18), R. F. Spee et al. (17), R. F. Spee et al. (19), S. C. Huang et al. (25)	2.57 [2.12, 3.02]	89	<0.00001	
R (W/R)						
>1	5	A. D. da Silveira et al. (21), R. F. Speeet al. (17), R. F. Spee et al. (19), S. Mueller et al. (16), S. S. Angadi et al. (23)	1.63 [0.04, 3.22]	0	=0.04	Chi^2^ = 1.66, df = 1 (*P* = 0.20)
≤1	5	C. H. Chou et al. (20), F. Besnier et al. (24), N. M. Benda et al. (18), N. Turri-Silva et al. (22), S. C. Huang et al. (25)	2.60 [2.15, 3.05]	90	<0.00001	

HF groups, heart failure disease groups; HIIT, high-intensity interval training; HIIT-L, protocol of HIIT with long work interval; HIIT-S, protocol of HIIT with short work interval; *I*^2^, heterogeneity; MD, mean difference; VO_2_peak, peak oxygen uptake; R (W/R), work-recovery ratio.

Certain studies were not included because they did not report the value used for subgroup analysis.

a Test for overall effect.

In conclusion, in patients with CAD, the overall training load of HIIT (including the number of sessions and total training period), single-session duration, and HIIT protocol are important factors influencing VO_2_peak. In patients with HF, single-session duration is the main influencing factor, while the number of sessions, total training period, HIIT frequency, interval duration, interval recovery, and HIIT protocol have relatively minor effects. Future research should further explore the individualized responses of different patient phenotypes to HIIT and the optimal exercise parameters.

### 4.6 Secondary outcomes

#### 4.6.1 Quality of life

In this systematic review, a total of 8 studies evaluated health-related quality of life. The assessment tools included the Minnesota Living with Heart Failure Questionnaire (MLHFQ), the 36-Item Short Form Health Survey (SF-36), the EuroQol Five-Dimensional Health Scale (EQ-5D), etc. Although there is evidence suggesting that HIIT may improve patients' quality of life, due to the heterogeneity of measurement tools and the negative results of some studies, a definitive conclusion cannot be drawn at present.

#### 4.6.2 Hospitalization

Among the 19 included studies, none specifically reported hospitalization rate as a pre-specified outcome. Therefore, based on the current evidence, the impact of HIIT on the hospitalization rate of patients with coronary artery disease or heart failure cannot be determined.

#### 4.6.3 Safety and adverse events

Safety is a key factor in the clinical application of HIIT. Among the 19 included studies, 9 reported that no serious adverse events (such as fatal arrhythmia, acute myocardial infarction) occurred during supervised HIIT. Mild to moderate adverse events (such as musculoskeletal discomfort, fatigue) were reported in some studies, but there was no significant difference in their occurrence frequency between the HIIT group and the moderate-intensity continuous training group. One study by G. McGregor et al. (29) reported 3 adverse events, which were considered possibly related to exercise but did not result in serious consequences. Overall, HIIT shows good safety for CAD and HF patients with stable conditions under a pre-set protocol with medical supervision.

### 4.7 Meta-regression analysis of the impact of exercise effort level evaluated based on peak RER on the improvement of VO_2_peak

To validate the potential mechanism underlying the influence of age on the improvement of VO_2_peak, we analyzed the role of exercise effort. Subgroup analysis suggested that the improvement in VO_2_peak among CAD patients aged ≤60 years might be greater than that among elderly patients (>60 years). We hypothesized that elderly patients might have difficulty achieving maximal effort during the test due to comorbidities, psychological or functional factors, leading to a systematic underestimation of VO_2_peak.

To this end, we employed meta-regression analysis to evaluate the relationship between the average age of patients and the peak respiratory exchange ratio (RER). The results showed a negative association trend between age and peak RER (β = −0.0053982), but it did not reach statistical significance (*P* = 0.249). This result supports the notion that elderly patients may have physiological or behavioral effort limitations, which can affect the accuracy of VO_2_ peak assessment (Table 7).

## 5 Publication bias assessment

**Figure 5:**
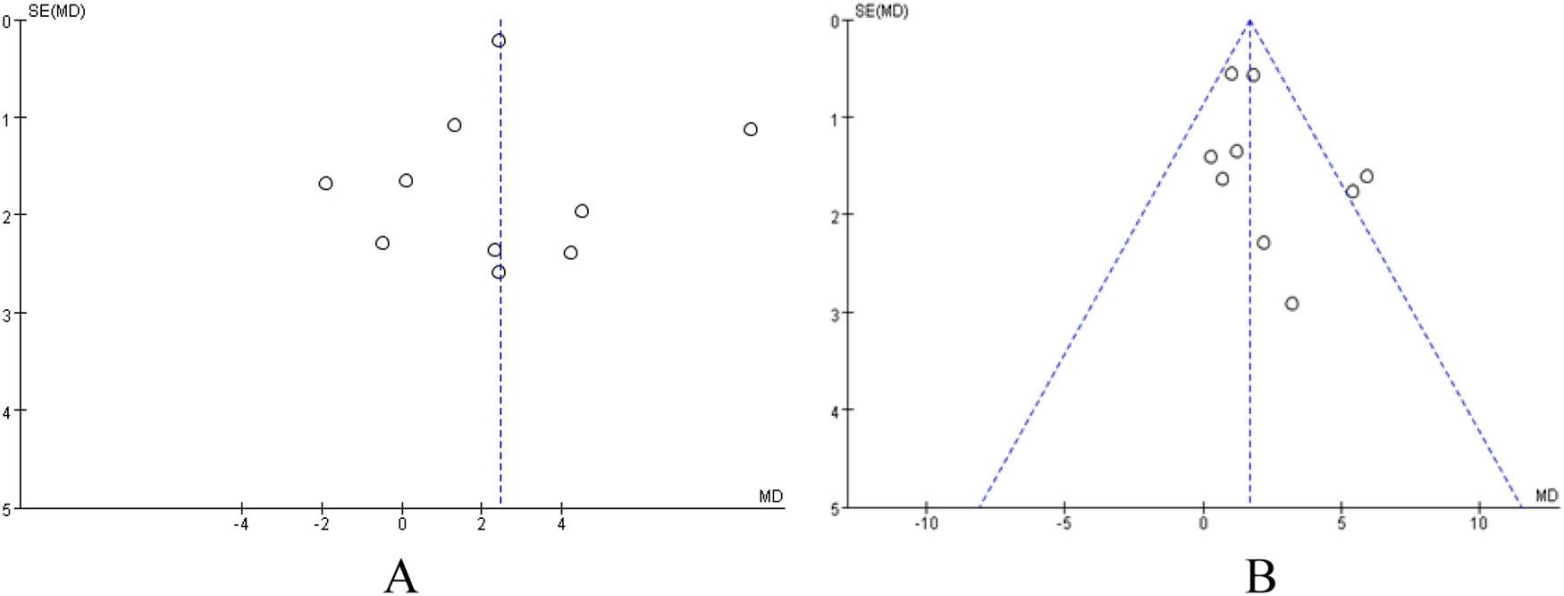
**(A)** funnel plot analysis of VO_2_peak in patients with heart failure. **(B)** Funnel Plot Analysis of VO_2_peak in patients with coronary artery disease.

To assess the potential publication bias and small-sample-size study effects in this Meta-analysis, we used Review Manager 5.4 software to draw funnel plots for analysis targeting the outcome indicator VO_2_peak in patients with coronary artery disease and heart failure, respectively. The results showed that in the VO_2_peak funnel plots of the two groups of patients, the study points were roughly symmetrically distributed on both sides of the pooled effect size estimate, and the overall shape was an inverted funnel (Figures 5A,B). Visual judgment did not indicate obvious signs of asymmetry.

**Figure 6:**
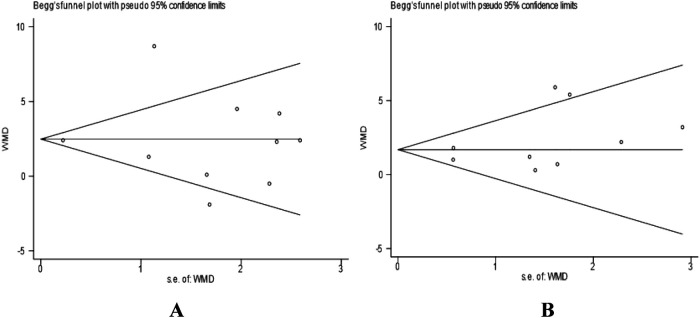
**(A)** Begg's funnel plot for studies on VO_2_peak in heart failure patients. **(B)** Begg's funnel plot for studies on VO_2_peak in coronary artery disease patients. Standard error was used as the precision measure. The dashed lines represent the pseudo 95% confidence limits.

In addition to visual inspection, we further employed Egger's test and Begg's test to quantitatively evaluate publication bias. In the analysis of VO_2_peak in heart failure patients, the results of Begg's test (*P* = 0.721) and Egger's test (*P* = 0.955) indicated no significant publication bias for this outcome indicator (*P* > 0.05). The results of Begg's test are shown in Figure 6A. Similarly, in the analysis of VO_2_peak in coronary artery disease patients, Begg's test (*P* = 0.251) and Egger's test (*P* = 0.252) suggested no significant publication bias for this outcome indicator (*P* > 0.05). The Begg's test plot is shown in Figure 6B.

## 6 Sensitivity analysis

**Figure 7:**
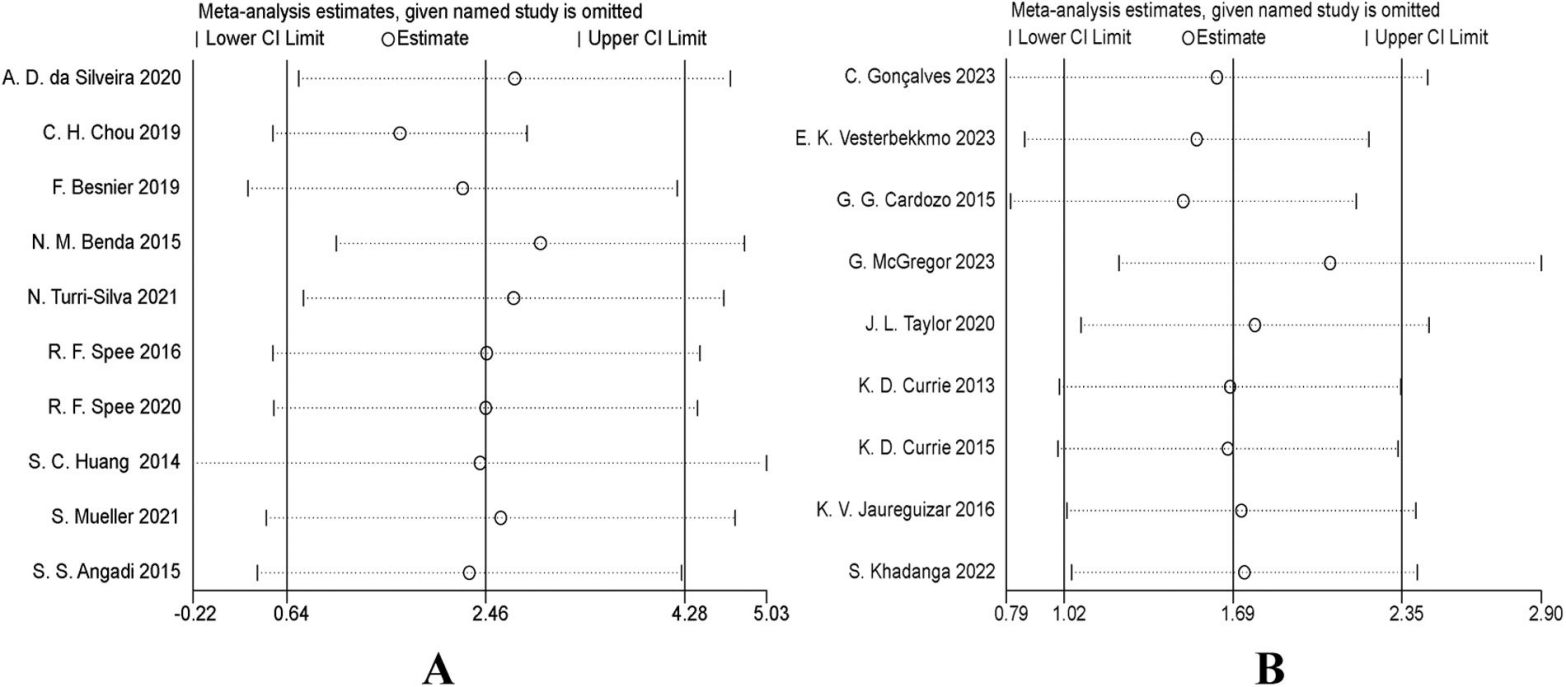
**(A)** sensitivity analysis forest plot: impact of sequentially removing each study on VO_2_peak pooled results (heart failure). **(B)** Sensitivity analysis forest plot: impact of sequentially removing each study on VO_2_peak pooled results (coronary artery disease).

To evaluate the robustness of the results of this Meta-analysis, we conducted a sensitivity analysis. Specifically, the “leave-one-out” method was adopted. After excluding each study one by one, the pooled effect size (VO_2_peak) was recalculated to observe its impact on the overall results. The results showed that in both the heart failure patient group and the coronary artery disease patient group, after excluding any single study item by item, the point estimate of the pooled effect size did not change significantly compared with the original overall estimate, and its 95% confidence interval overlapped with the original interval without directional reversal. We also conducted a sensitivity analysis on the quality of the included studies. After excluding studies with lower methodological quality scores, the pooled results remained stable. In conclusion, the results of the sensitivity analysis indicate that the results of this Meta-analysis are highly robust and are less affected by individual studies or low-quality studies, which enhances the reliability of the conclusions (Figures 7A,B).

## 7 Discussion

This study comprehensively evaluated the effects of high-intensity interval training (HIIT) at different doses on the peak oxygen uptake (VO_2_peak) in patients with coronary artery disease (CAD) and heart failure (HF) through a systematic review and meta-analysis, and deeply explored the moderating effects of population characteristics and exercise parameters on the intervention outcomes. In addition, this study for the first time comprehensively evaluated the benefits of HIIT on the submaximal exercise index, oxygen uptake at the first ventilatory threshold (VO_2_ at VT1), through a meta-analysis. The research results provide important evidence for further optimizing the individualized dose of HIIT in cardiac rehabilitation. The results are comprehensively analyzed and discussed from multiple dimensions below.

### 7.1 Overall effectiveness of HIIT on VO_2_peak in patients with CAD and HF

High-intensity interval training (HIIT) can significantly improve the peak oxygen uptake (VO_2_peak) in patients with coronary artery disease (CAD) and heart failure (HF), with the overall effect sizes being +1.69 mL·kg^−1^·min^−1^ (95% CI: 1.02–2.35) for CAD patients and +2.46 mL·kg^−1^·min^−1^ (95% CI: 0.64–4.28) for HF patients, which is consistent with the conclusions of previous studies (35–39). Although the improvement in HF patients is numerically greater, it has high heterogeneity (*I*^2^ = 79%), reflecting the clinical diversity of this population and the differences in research methodologies. As the gold standard for cardiopulmonary function assessment, the improvement of VO_2_peak may be limited by the cardiopulmonary reserve of HF patients; in recent years, some viewpoints suggest that VO_2_ atVT1 may more sensitively reflect the improvement of submaximal exercise endurance in HF patients (12).

Compared with previous studies (40–43), this study not only validates the positive effects of HIIT on two types of patients but also highlights the significant heterogeneity among HF patients. Previous studies mainly focused on the comparison between HIIT and MICT, and the exploration of how factors such as disease phenotypes, baseline cardiac function, and individual tolerance affect the dose-response of HIIT was relatively limited.

Based on the confirmation of the overall effectiveness of HIIT, this study revealed the characteristics of efficacy heterogeneity in HF patients, suggesting that in clinical practice, it is necessary to formulate individualized exercise prescriptions by considering multi-dimensional factors such as disease phenotypes, cardiac function classifications, comorbidities, and tolerance, thus providing a basis for promoting precise cardiac rehabilitation.

### 7.2 Modulatory effects of population characteristics on the dose-response relationship of HIIT

Population characteristics significantly modulate the dose-response relationship of HIIT, and there are disease-specific differences.

In patients with CAD, studies with a sample size of ≥14 showed robust improvement, while the results of small-sample studies were unstable. This trend was more pronounced in patients with HF. The effect size was higher in the large-sample group, and the difference between groups reached statistical significance (*P* = 0.01), indicating that sample size is an important modifier.

The regulatory effect of age is disease-specific: in patients with coronary artery disease (CAD), the group aged ≤60 years benefits more significantly, while in patients with heart failure (HF), the group aged >60 years shows more stable improvement. Meta-regression analysis suggests that the better improvement of VO_2_peak in young CAD patients may be partly due to the underestimation of measurement caused by insufficient test efforts in the elderly group (there is a negative correlation trend between age and peak RER, β = −0.0053982). The methodological quality has a significant impact on the effect estimation. In both types of populations, studies with a score ≤7 show larger effect sizes, indicating that high-quality studies provide more conservative and reliable estimates.

Compared with previous studies (44–46), this study systematically elaborated the regulatory differences in multiple population characteristics for the first time, providing a new basis for individualized dose setting. These findings emphasize that characteristics such as age and disease type need to be comprehensively considered when formulating HIIT prescriptions, and the influence of sample size and methodological quality should be fully controlled in the study design.

### 7.3 Modulatory effects of exercise dose parameters on HIIT outcomes and their disease-specific differences

Exercise dose parameters have a significant regulatory effect on the improvement of VO_2_peak by HIIT, and there are disease-specific differences.

In patients with CAD, the total training volume is the core influencing factor: Subgroups with Number of sessions ≥36 sessions, Total Training Period ≥12 weeks, and Single-session Duration >35 min showed more significant improvements in VO_2_peak (all *P* < 0.05), establishing a clear dose-response relationship. HIIT frequency (3–4 days/week vs. ≤2 days/week), work/recovery ratio, Type of recovery, and HIIT Protocol (HIIT-L vs. HIIT-S) did not show significant intergroup differences.

In patients with heart failure (HF), a single-session duration of >35 min is the only dose parameter that consistently shows significance. Regarding the high-intensity interval training (HIIT) frequency, 3–4 days/week shows clear benefits, while ≤2 days/week leads to limited improvement. Although other parameters, such as interval recovery, type of recovery, and work/rest ratio (W/R), do not show statistical significance, the high heterogeneity among groups suggests that the dose-response relationship in HF patients is more complex (47).

Compared with previous studies, this research supports the conclusions of Guizhen Hong et al. (48) and Zhi-Jian Wu et al. (49) regarding the central role of the total training volume. However, it particularly emphasizes the independent influence of training frequency in patients with heart failure (HF), providing new evidence for the existing theory. Meanwhile, this study reveals the potential sources of the efficacy heterogeneity reported by Haohan Yu et al. (46) from the perspective of dosage parameters.

The results indicate that the rehabilitation outcomes of patients with coronary artery disease (CAD) primarily depend on the accumulation of total training load, while patients with heart failure (HF) are more sensitive to single-session duration and HIIT frequency. This finding supports the differentiation of disease types when formulating HIIT prescriptions: for CAD rehabilitation, ensuring the total training volume should be prioritized, and for HF rehabilitation, the configuration of single-session dose and frequency needs to be optimized simultaneously.

### 7.4 Mechanism exploration: potential reasons for the significant yet highly heterogeneous improvement in VO_2_peak among HF patients

After high-intensity interval training (HIIT) in patients with heart failure (HF), the peak oxygen uptake (VO_2_peak) was significantly improved (MD = +2.46 mL·kg^−1^·min^−1^), but there was high heterogeneity (*I*^2^ = 79%), indicating significant differences in the efficacy of this population.

Previous studies (40–43) have confirmed the effectiveness of HIIT in patients with HF, but there is insufficient exploration of the mechanisms underlying the variability of its efficacy. Through in-depth analysis in this study, it was found that the heterogeneity mainly stems from the following aspects:

The inherent differences in the phenotypic manifestations of heart failure (HF) represent a pivotal factor. There are fundamental disparities in the pathological mechanisms and exercise physiological responses between patients with heart failure with reduced ejection fraction (HFrEF) and those with heart failure with preserved ejection fraction (HFpEF) (50, 51). Central adaptations induced by high-intensity interval training (HIIT), such as an increase in cardiac output, may be more pronounced in HFrEF patients, while peripheral adaptations, such as improved oxygen utilization in skeletal muscles, could be the primary pathway for functional improvement in HFpEF patients (52, 53). Failure to adequately distinguish between HF phenotypes in research can introduce significant variability.

Differences in drug treatment and exercise-drug interactions are crucial (54). Most patients with heart failure (HF) receive individualized multi-drug therapies such as β—β-blockers and SGLT2 inhibitors (55). The effects of these drugs on heart rate, metabolism, etc., may regulate the direction and intensity of patients' responses to exercise training.

In conclusion, the substantial improvement potential and high heterogeneity exhibited by HF patients during HIIT intervention are the complex outcomes of the combined effects of their disease phenotypes, medication backgrounds, individual tolerances and compliance, as well as differences in research methodologies. Future research should focus on exploring the individualized HIIT dose-response relationship in more homogeneous HF subgroups (e.g., strictly distinguishing between HFpEF and HFrEF) and with full consideration of the medication background, so as to promote the effective and safe application of HIIT in the precision rehabilitation of HF (56, 57).

### 7.5 Clinical significance and practical recommendations

In clinical practice, patients with coronary artery disease (CAD) should focus on increasing total training volume and extending the duration of each session. Conversely, heart failure (HF) patients should emphasize training frequency and session duration over total volume, while carefully monitoring individual responses during interventions. When designing a high-intensity interval training (HIIT) program, factors such as frequency, session duration, recovery intensity, and work/recovery ratio must be considered comprehensively. Thus, HIIT prescriptions should be individualized. Based on our study results, we recommend the following HIIT dosages (Table 8):

**Table 7:** Summary of meta-regression results for the effect of age on peak RER following cardiac rehabilitation.

Variable	Coefficient	Std. Err.	*t*-value	*p*-value	95% Conf. interval
Mean Age	−0.0053982	0.0037841	−1.43	0.249	[−0.0174408, 0.0066444]
Constant	0.3457885	0.2366413	1.46	0.240	[−0.4073097, 1.098887]

**Table 8:** Recommendations on HIIT protocol for HF and CAD patients.

Disease	Frequency, days/week	Total Training Period program, weeks	Single-session duration, min	Intensity of recovery, VO_2_peak, %	Ratio, work/recovery
CAD	≥2 (2–5)	≥12 (3.5–12)	>35 (20–50)	≥40% (35%–60%)	0.5–1.33
HF	≥3 (2–3)	≥12 (4–24)	>35 (20–40)	≥40% (10%–70%)	0.5–1

CAD, coronary artery disease; HF, heart failure; HIIT, high-intensity interval training groups; VO_2_peak: peak oxygen uptake.

To facilitate direct clinical application, we further developed a template for an 8–12-week structured HIIT rehabilitation program based on Table 8 (see Table 9). This template encompasses the specific warm-up-main exercise-cool-down structure, intensity monitoring methods, and individualized adjustment suggestions, which can be used as a reference for cardiac rehabilitation physicians, physical therapists, and exercise physiologists in their actual work.

**Table 9:** Clinical implementation template: 8- to 12-week HIIT rehabilitation program for CAD and HF patients.

Disease type	Sessions per week	Total training period	Sample session structure	Intensity monitoring	Notes & adjustment recommendations
CAD (Coronary Artery Disease)	3–4 sessions/week	8–12 weeks	**Warm-up:** 5–10 min; **HIIT Main Body:** 4–6 sets × 3–4 min (85%–95%HR_peak_); **Recovery:** 2–3 min (40%–60%VO_2_peak; **Cool-down:** 5 min	Use a heart rate monitor or Rating of Perceived Exertion (RPE 15–17)	Can incorporate aerobic equipment (treadmill, stationary bike). If tolerated well, gradually increase the number of sets or session duration.
HF (Heart Failure)	3 sessions/week	12 weeks	**Warm-up:** 10 min; **HIIT Main Body:** 4–5 sets × 2–4 min (80%–90%HR_peak_); **Recovery:** 2–3 min (≤40% VO_2_peak) **Cool-down:** 5–10 min	Closely monitor blood oxygen saturation (SpO₂) and symptoms. Initial sessions recommended under supervision.	Stop immediately if shortness of breath, chest pain, or SpO₂ < 90% occurs. Consider combining with inspiratory muscle training to improve tolerance.

HR_peak_: Peak Heart Rate, recommended to be set based on results from a symptom-limited cardiopulmonary exercise test (CPET).

RPE: Borg Rating of Perceived Exertion.

SpO_2_: Peripheral capillary oxygen saturation.

All patients must undergo clinical evaluation and risk stratification before starting the program.

Functional assessment recommendations: For patients with heart failure (HF), in addition to routine monitoring of VO_2_peak, rehabilitation centers with appropriate conditions may consider simultaneously evaluating the changes in the first ventilatory threshold (VO_2_ at VT1) through cardiopulmonary exercise testing (CPET) to more sensitively detect the early improvement in their submaximal exercise endurance.

### 7.6 Limitations of this study

The reporting of secondary outcome indicators is incomplete. The number of studies relying on VO_2_ at VT1 is less than that of studies reporting VO_2_peak, which may reduce the statistical power and generalizability of this specific finding. There is high heterogeneity in the results, and there are risks of implementation and measurement biases in the original studies. There is a lack of long-term follow-up data. There is a confounding of effort levels. Although it has been confirmed that age is negatively correlated with peak RER, the root cause of insufficient effort in elderly patients remains unclear.

### 7.7 Future research directions

Enhance the evaluation by adopting standardized indicators and systematically monitoring VO_2_ at VT1 for a more sensitive assessment of endurance improvement. Strengthen the evidence by conducting high-quality long-term follow-up RCTs to clarify the long-term benefits and optimal dosage of HIIT. In homogeneous subgroups, use the peak RER as a covariate to accurately evaluate individual physiological benefits.

## 8 Conclusions

This study demonstrated that high-intensity interval training (HIIT) can effectively improve the peak oxygen consumption (VO_2_peak) in patients with coronary artery disease (CAD) and heart failure (HF), and a greater improvement potential was observed in HF patients. Through dose-response analysis, this study for the first time identified disease-specific optimal HIIT parameters for patients with CAD and HF, providing a crucial evidence-based basis for formulating individualized and precise HIIT exercise prescriptions in clinical practice. Future studies should validate these dose-response relationships in a larger population and further explore the impact of other variables in HIIT programs (such as exercise mode and intensity during the work period) on clinical outcomes. Moreover, it is strongly recommended that future functional assessments go beyond the single indicator of VO_2_peak and incorporate more sensitive submaximal exercise endurance indicators, such as VO_2_ at VT1, into routine considerations. This will contribute to a more comprehensive and accurate evaluation of the improvement effect of HIIT on patients’ daily functional status and further optimize the exercise rehabilitation framework for patients with chronic heart diseases.
