# Supplementary material for: Optimal doses of high-intensity interval training in patients with coronary artery disease and heart failure: a systematic review and meta-analysis
Source: Front Cardiovasc Med. 2026 Jan 2;12:1698310. doi: 10.3389/fcvm.2025.1698310 (PMC12829331; doi:10.3389/fcvm.2025.1698310)
Supplement: Supplementary file 1 [file Datasheet1.pdf]

Search Strategy

Electronic searches were conducted in the MEDLINE, CINAHL, Web of Science, PubMed, Cochrane Library, Embase, and EBSCO databases for all English publications published before September 2025. The Medical Subject Headings (MeSH) database was used to identify all relevant articles concerning HIIL and coronary artery disease (CAD) or heart failure (HF). The MeSH terms employed were "Coronary Artery Disease" OR "Heart Failure" OR "Cardiac Failure" OR "Heart Decompensation" AND "Randomized Controlled Trial" along with their related terms. The text words used in combination with MeSH terms were "High-Intensity Interval Training" OR "Interval Training, High-Intensity" OR "Trainings, High-Intensity Interval."

Search Methods

PubMed

((("coronary artery disease"[MeSH Terms] OR ("coronary"[All Fields] AND "artery"[All Fields] AND "disease"[All Fields]) OR "coronary artery disease"[All Fields] OR ("heart failure"[MeSH Terms] OR ("heart"[All Fields] AND "failure"[All Fields]) OR "heart failure"[All Fields]) OR ("heart failure"[MeSH Terms] OR ("heart"[All Fields] AND "failure"[All Fields]) OR "heart failure"[All Fields] OR ("cardiac"[All Fields] AND "failure"[All Fields]) OR "cardiac failure"[All Fields]) OR ("heart failure"[MeSH Terms] OR ("heart"[All Fields] AND "failure"[All Fields]) OR "heart failure"[All Fields] OR ("heart"[All Fields] AND "decompensation"[All Fields]) OR "heart decompensation"[All Fields])) AND ("high intensity interval training"[MeSH Terms] OR ("high intensity"[All Fields] AND "interval"[All Fields] AND "training"[All Fields]) OR "high intensity interval training"[All Fields] OR ("high"[All Fields] AND "intensity"[All Fields] AND "interval"[All Fields] AND "trainings"[All Fields]) OR "high intensity interval trainings"[All Fields])) OR ("high intensity interval training"[MeSH Terms] OR ("high intensity"[All Fields] AND "interval"[All Fields] AND "training"[All Fields]) OR "high intensity interval training"[All Fields] OR ("interval"[All Fields] AND "training"[All Fields] AND "high"[All Fields] AND "intensity"[All Fields]) OR "interval training high intensity"[All Fields]) OR ("high intensity interval training"[MeSH Terms] OR ("high intensity"[All Fields] AND "interval"[All Fields] AND "training"[All Fields]) OR "high intensity interval training"[All Fields] OR ("trainings"[All Fields] AND "high"[All Fields] AND "intensity"[All Fields] AND "interval"[All Fields]))) AND (randomizedcontrolledtrial[Filter])

Web of Science

“High-Intensity Interval Trainings” OR “High Intensity Interval Training” OR “Interval

Training, High-Intensity" OR "High-Intensity Intermittent Exercise" OR "Trainings, High-Intensity Interval"AND"Coronary Artery Disease"OR"Heart Failure" OR "Cardiac Failure" OR"Heart Decompensation"AND"Randomized Controlled Trial"

Cochrane Library

#1

MeSH descriptor: [High-Intensity Interval Training] explode all trees MeSH 1270

#2

(High Intensity Interval Training):ti,ab,kw OR (Interval Training, High-Intensity):ti,ab,kw OR (High-Intensity Intermittent Exercise):ti,ab,kw OR (Trainings, High-Intensity Interval):ti,ab,kw Limits 5108

#3

#1 OR #2 Limits 5108

#4 Coronary artery disease Limits 29294

#5 MeSH descriptor: [Heart Failure] explode all trees MeSH 14955

#6 (Cardiac Failure):ti,ab,kw OR (Heart Decompensation):ti,ab,kw Limits 20805

#7

#5 OR #6 Limits 30296

#8

#4 OR #7 Limits 56842

#9

Randomized Controlled Trial Limits 1161812

#10

#3 AND #8 AND #9 Limits 199

Embase

#6#1 AND #4 AND #5

#5'randomized controlled trial':ab,ti 202,573

#4 #2 OR # 3590,078

#3'coronary artery disease':ab,ti 180,311

#2'heart failure':ab,ti OR 'cardiac failure':ab,ti OR 'heart decompensation':ab,ti 433,295

#1 ('high-intensity interval trainings':ab,ti OR 'high intensity interval training':ab,ti OR 'interval training, high-intensity':ab,ti) AND 'trainings, high-intensity interval':ab,ti

EBSCO/MEDLINE/CINAHL

"High-Intensity Interval Trainings" or "Interval Training, High-Intensity" or "High-Intensity Intermittent Exercise" or "Trainings, High-Intensity Interval"

AND "Coronary Artery Disease" or "Heart Failure" or "Cardiac Failure" or "Heart Decompensation"

AND "Randomized Controlled Trial"
